# Supplementary material for: Thermodynamics-guided alloy and process design for additive manufacturing
Source: Nat Commun. 2022 Jul 27;13:4361. doi: 10.1038/s41467-022-31969-y (PMC9329330; doi:10.1038/s41467-022-31969-y)
Supplement: Supplementary file 1 — Supplementary Information [file 41467_2022_31969_MOESM1_ESM.docx]

**Supplementary Information**

**Thermodynamics-guided alloy and process design for additive manufacturing**

Zhongji Sun^a,b^*, Yan Ma^a^, Dirk Ponge^a^, Stefan Zaefferer^a^, Eric A. Jägle^c^, Baptiste Gault^a,d^, Anthony D. Rollett^e^, Dierk Raabe^a^

a. Department of Microstructure Physics and Alloy Design, Max-Planck-Institut für Eisenforschung GmbH, Max-Planck-Straße 1, 40237, Düsseldorf, Germany

b. Institute of Materials Research and Engineering, A*STAR (Agency for Science, Technology and Research), 138634 Singapore

c. Institute of Materials Science, Universität der Bundeswehr München, 85579 Neubiberg, Germany

d. Department of Materials, Royal School of Mines, Imperial College London, London, SW7 2AZ, United Kingdom

e. Department of Materials Science and Engineering, Carnegie Mellon University, Pittsburgh, PA, 15213-3890, USA

* Corresponding author: z.sun@mpie (Zhongji Sun)


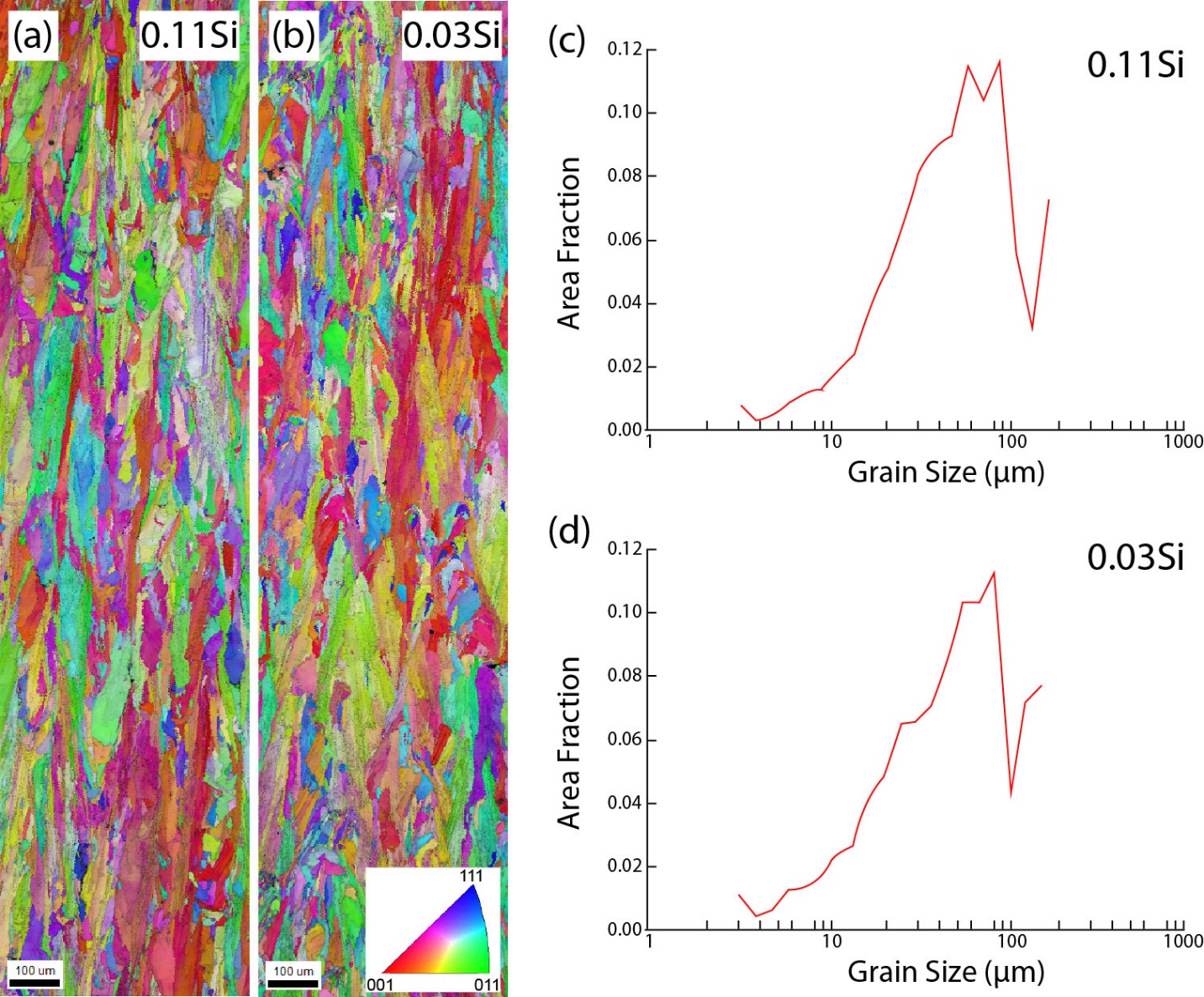


**Supplementary Fig. 1. EBSD measurement of the 0.11Si and 0.03Si samples along the build direction. a,b** Inverse pole figure maps of the 0.11Si and 0.03Si sample viewed perpendicular to the build direction. **c,d** the grain size distributions for the 0.11Si and 0.03Si samples show every similar trend.


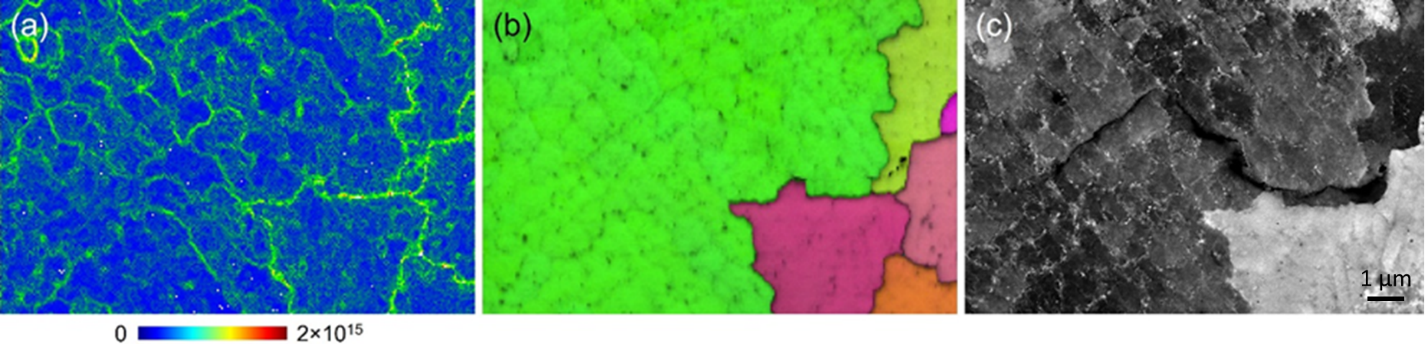


**Supplementary Fig. 2. Geometrically Necessary Dislocations (GNDs) mapping for interdendritic hot cracks in the 0.11Si alloy. a** GND map overing the interdendritic hot crack in the as-built 0.11Si sample. **b,c** The IPF map and the ECCI map of the same region.

As shown by the geometrically necessary dislocations (GNDs) mapping in Supplementary Fig. 2(a), no obvious crack propagation path can be observed. This is because the GNDs map is only effective for examining elastic stress states within the material. In the current case, during the crack initiation and propagation, plastic deformations mostly occurred. Thus, most residual stresses captured by the GNDs map concentrate at the grain boundary regions when comparing to Supplementary Fig. 2(b). Only the ECCI map can truly reflect the size and morphology of the interdendritic hot crack in this regard (Supplementary Fig. 2(c)).


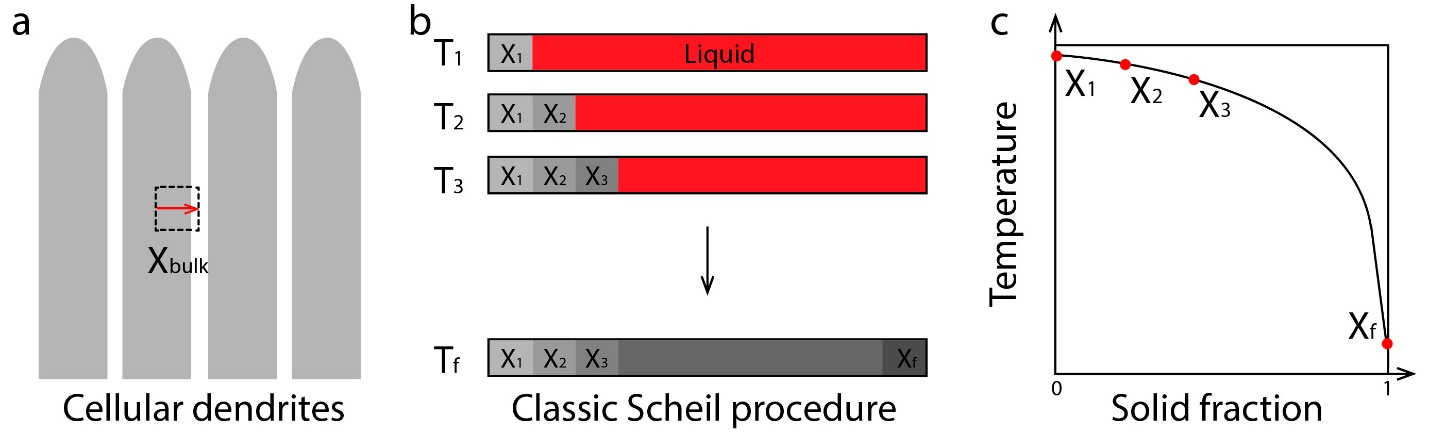


**Supplementary Fig. 3. Calculation procedure for the classical Scheil model. a** The classical Scheil model computes the composition change across the dendritic and interdendritic regions during solidification. **b** It is composed of many equilibrium calculations controlled by stepwise temperature decrease. **c** The typical solidification range curve is then constructed with the temperature and solid fraction information from the equilibrium calculations.

(1) The principles of a classical Scheil calculation

Scheil simulations essentially calculate the change in composition during solidification, across the dendritic/interdendritic regions along the red arrow shown in Sup. Fig. 2(a). The overall composition containing half of the dendritic and half of the interdendritic regions (circled in dotted black rectangle) must be the same as the bulk alloy composition.

This means that such Scheil simulations are composed of many stepwise equilibrium calculations, as shown in Sup. Fig. 2(b). In each of the consecutive simulation steps, the temperature is gradually – step-by-step – lowered by a fixed amount, and for these fixed boundary conditions an equilibrium calculation is then performed for his very step, to compute the composition and molar fraction of the solid/liquid phases. For instance, at temperature T_1_, the equilibrium calculation results in a solid composition of X_1_. At temperature T_2_, the remaining liquid from temperature T_1_ goes through another equilibrium calculation. A fixed amount of solid with composition X_2_ is thus yielded in this step. Such equilibrium calculations will continue until only a small portion of liquid (typically set as 0.01 fraction of the initial liquid volume fraction) is retained in the system. The overall bulk composition, X_bulk_, must be equal to the average of all individual solid compositions, X_1_ + X_2_ + X_3_ + ··· + X_f_.

By plotting the solid fraction information against temperature during each calculation step, we can obtain the typical solidification range graph as shown in Sup. Fig. 2(c). Due to this calculation scheme, the accuracy of the solidification interval predicted by the classical Scheil simulation is heavily dependent on the precision of elemental partitioning information computed in Sup. Fig. 2(b).

(2) Limitations of Scheil calculation for the case of rapid solidification

Under the condition of rapid solidification for AM processing, the classical Scheil simulation fails to predict the partitioning information shown in Sup. Fig. 2(b) with sufficient accuracy. This is mainly because the Scheil model rests on 3 major assumptions. First, the Scheil model assumes equilibrium partitioning at the solid/liquid interface. Second, there is infinitely fast diffusion assumed in the liquid. Third, there is zero diffusion assumed in the solid. However, during the actual rapid solidification process, these assumptions (especially the first 2) are not describing the situation well enough.

The fast solid/liquid interface velocity will trap slow diffusing elements within the solid before they can partition into the liquid, an effect which is commonly termed “solute trapping”. Moreover, the partitioned solutes in the liquid do not have sufficient time to diffuse away. This pile-up of solutes ahead of the solidification front will then create a constitutional undercooling. Both effects will reduce the prediction accuracy for the scenario shown in Sup. Fig. 2(b). As explained before, when this information is incorrect, the predicted solidification range under these Scheil conditions will also be flawed. Since the solidification interval is often used to predict the hot cracking susceptibility of an alloy, this will unavoidably yield non-accurate predictions.

(3) Advantage of our method compared to the classical Scheil model

In the approach used in this paper, we resolve this problem by directly using the precise near-atomic-scale chemical information across the dendritic/interdendritic regions, as revealed by APT. Each APT data point corresponds to a single solid composition in Sup. Fig. 2(b) (e.g., X_1_, X_2_, and X_3_, etc.). Their respective solidus temperatures are thus the same as those pre-defined temperatures in the classical Scheil model (e.g., T_1_, T_2_ and T_3_, etc.), which can be obtained through local equilibrium calculations, using this local composition information. Therefore, the current method is an improvement over the classical Scheil approach, due to the availability of the actually measured chemical partitioning information, i.e., its direct coupling with experimental data.


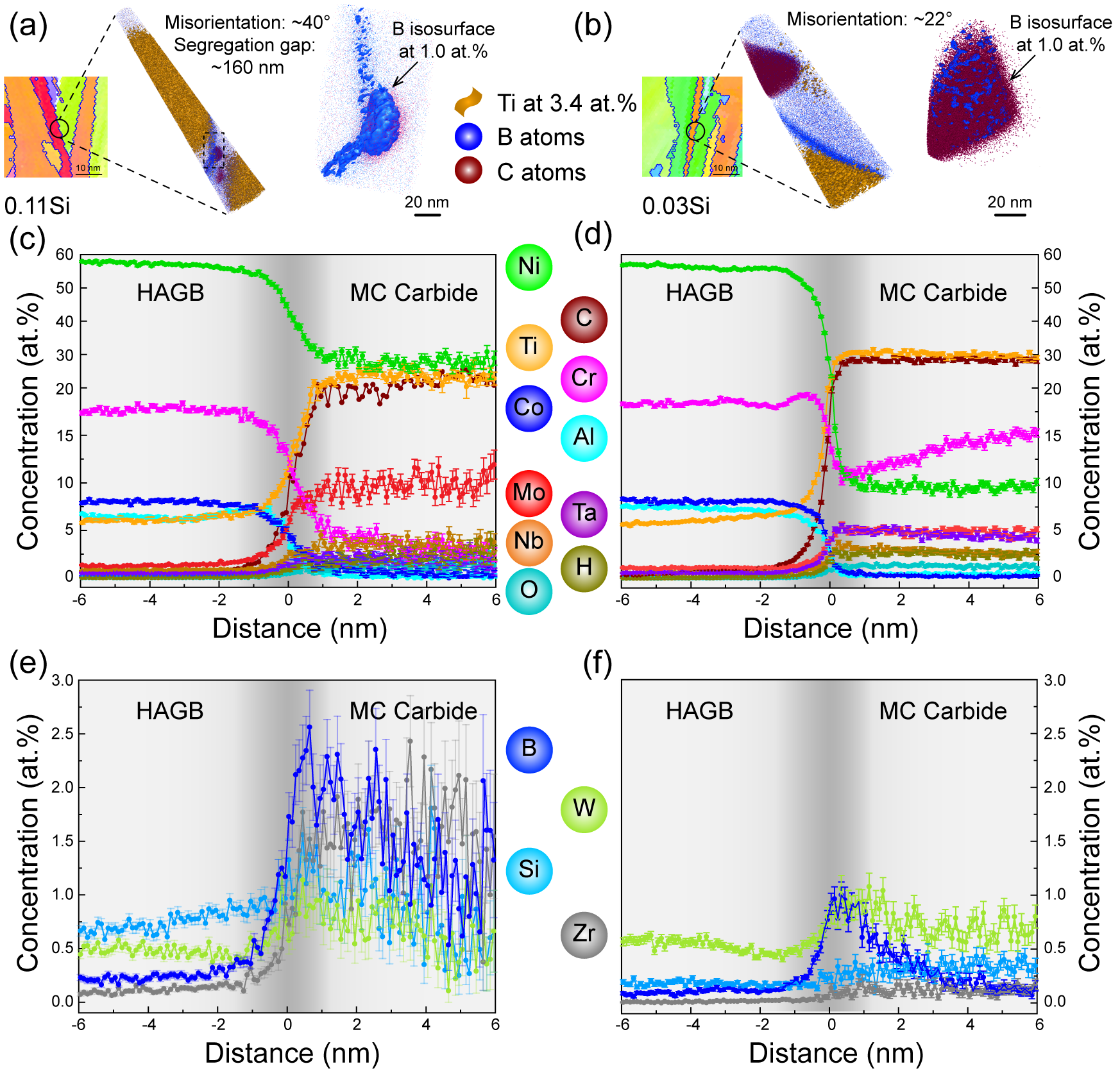


**Supplementary Fig. 4. APT measurements of the HAGBs for the 0.11Si and 0.03Si samples. a,b** Both samples are extracted from the HAGBs. Despite the presence of carbides, B segregation is still present for both materials. The 0.11Si sample’s carbide is smaller compared to the 0.03Si’s counterpart. **c,d** Proximity diagrams for the major elements within the carbides are displayed. The carbide in the 0.11Si sample’s HAGB has a lower Ti/C concentration of ~20 at.%. The C/Ti amount in the 0.03Si sample’s HAGB is ~30 at.%, similar to those interdendritic carbides. **e,f** B is still segregating at the interphase interfaces like the interdendritic counterparts.

**Supplementary Table 1.** Dendritic compositions measured by the APT experiments for three samples investigated in this work.

| **Sample** | **Cr** | **Co** | **Al** | **Ti** | **Mo** | **W** | **Nb** | **Ta** | **Si** | **Zr** | **C** | **B** | **Ni** |
| --- | --- | --- | --- | --- | --- | --- | --- | --- | --- | --- | --- | --- | --- |
| **0.11Si (110 W)** | 17.13 ±  0.42 | 8.88 ± 0.79 | 6.97 ± 0.25 | 3.62 ± 0.03 | 1.00 ± 0.09 | 0.72 ± 0.05 | 0.37 ± 0.05 | 0.62 ± 0.23 | 0.29 ± 0.01 | 0.008 ± 0.003 | 0.057 ± 0.012 | 0.041 ± 0.011 | 60.33 ±  1.27 |
| **0.11Si (185 W)** | 17.51 ±  0.28 | 8.62 ± 0.37 | 6.85 ± 0.17 | 3.46 ± 0.04 | 1.01 ± 0.08 | 0.69 ± 0.04 | 0.36 ± 0.03 | 0.58 ± 0.12 | 0.27 ± 0.01 | 0.009 ± 0.003 | 0.041 ± 0.002 | 0.036 ± 0.016 | 60.59 ±  0.55 |
| **0.03Si (185 W)** | 17.39 ±  0.16 | 8.49 ± 0.11 | 7.40 ± 0.07 | 3.42 ± 0.02 | 0.95 ± 0.07 | 0.67 ± 0.02 | 0.30 ± 0.01 | 0.48 ± 0.01 | 0.13 ± 0.01 | 0.005 ± 0.002 | 0.043 ± 0.003 | 0.045 ± 0.011 | 60.73 ±  0.31 |
